# Gene expression profiling and functional analysis reveals that p53 pathway-related gene expression is highly activated in cancer cells treated by cold atmospheric plasma-activated medium

**DOI:** 10.7717/peerj.3751

**Published:** 2017-08-25

**Authors:** Lei Shi, Lihua Yu, Fagui Zou, Huimin Hu, Kun Liu, Zhenghong Lin

**Affiliations:** 1 School of Life Sciences, Chongqing University, Chongqing, PR China; 2 The State Key Laboratory of Power Transmission Equipment & System Security and New Technology, Chongqing University, Chongqing, PR China

**Keywords:** Plasma, Cancer therapy, Plasma activated medium, p53 pathway-related gene

## Abstract

**Background:**

Cold atmospheric-pressure plasma (CAP) has been considered a promising strategy for anti-cancer treatment. Traditionally, CAP was employed to kill cancer cells or tumor tissues by direct irradiation. However, CAP has some disadvantages such as infiltration capacity and storage convenience. Recently, plasma-activated medium (PAM) was used as an alternative strategy to treat cancer cells or tumors. The novel PAM approach has potential as an anti-cancer therapy.

**Objective:**

To reveal the global activation of signaling pathways in oral cancer cells induced by PAM.

**Methods:**

Oral squamous cell line SCC15 were treated by PAM and gene expression profiles were evaluated by using RNA-seq. Functional analyses were employed to reveal the global responses of SCC15 cells with PAM stimulation. QRT-PCR and Western blot were carried out to validate the expression levels of selected genes.

**Results:**

More than 6G clean data per sample were obtained in PAM-treated SCC15 cells. A total of 934 differentially expressed genes (DEGs) were identified and GO analysis implicated the deep involvement of biological process. KEGG mapping further clustered 40 pathways, revealing that “p53 pathway” was significantly enriched. SCC15 cells were commonly used as a p53-null cell line. Therefore, the enriched p53 pathway-related genes in our analysis might be activated by other stimulators, in a p53-independent manner. Gene set enrichment analysis (GSEA) was also performed to evaluate changes at the gene-sets level. The results demonstrated not only the high engagement of “p53 pathway” but also the involvement of novel pathways such as hypoxia pathway.

**Conclusions:**

The present study elucidates the transcriptomic changes of PAM treated SCC15 cells, containing highly enriched DEGs involved in “p53 pathway”. Our analysis in this work not only provides genomic resources for future studies but also gives novel insights to uncover the molecular mechanism of PAM stimulation.

## Introduction

Plasma is an ionized gas composed of ions, electrons, free radicals, and positive or negative charged particles. Over the last decade, the field of plasma medicine had been extensively investigated. Cold atmospheric pressure plasma (CAP), operated at near room temperature and atmospheric pressure, is particularly suited for the purpose of anti-cancer therapy (Hirst et al., 2016; Yan, Sherman & Keidar, 2017).

There is evidence from the recent literature to suggest that CAP shows a strong anti-cancer effects in a wide range of cancer cell lines, including melanoma, oral carcinoma, breast cancer, lung cancer and myeloma (Fridman et al., 2007; Kim et al., 2010a; Kim et al., 2010b; Kim et al., 2010c; Chang et al., 2014; Adachi et al., 2015; Vermeylen et al., 2016; Xu et al., 2016). Moreover, CAP has shown the ability to suppress or eradicate subcutaneous xenograft tumors in several mouse models (Yajima et al., 2014; Mirpour et al., 2016). Generally, the anti-tumor effect of CAP was predominantly attributed to the oxidative damage against the cancer cells (Vandamme et al., 2012; Ahn et al., 2014).

Two general CAP treatment approaches have been well documented. One is to employ the CAP to directly irradiate tumor tissues or cancer cells. Another strategy was developed in the last few years. Aqueous solutions were exposed to atmospheric plasma, generating cold plasma-activated medium (PAM) (Tanaka et al., 2011; Adachi et al., 2015; Yan et al., 2015; Boehm et al., 2016; Chen et al., 2016). By incubating with cancer cells or directly injected into the tissues, PAM can effectively kill tumor cells or tissues as the CAP direct irradiation does (Yan et al., 2015; Utsumi et al., 2014; Tanaka et al., 2016), hence PAM has great advantages. PAM can be readily generated, stored and maintain its anti-cancer activity for a relatively long period (Adachi et al., 2015; Judée et al., 2016). In addition, PAM can be injected directly into tumor tissues, avoiding the disadvantages of CAP penetration.

Direct CAP irradiation generates reactive, short-lived species, while PAM usually contains relatively long-lived secondary products, including hydrogen peroxide, nitrates and nitrites. Although differences still exist, it is thought that both approaches showed anti-tumor effects depending on reactive species (Bekeschus et al., 2014; Ahn et al., 2014). The study of CAP will also facilitate the understanding of PAM. CAP treatment causes an increase of intracellular ROS and up-regulation of the antioxidant system. Subsequently, the cell cycle is ceased and apoptosis initiates (Volotskova et al., 2012; Ishaq, Evans & Ostrikov, 2014). The key player in this battle is proposed to be H_2_O_2_ (Bekeschus et al., 2014). During this process, normal cells are more resistant to CAP treatment. Compared to normal cells, cancer cells usually generate high levels of ROS. Therefore, CAP has shown a selective killing capacity against cancer cells, while normal cells usually exhibit resistance to CAP (Keidar et al., 2011; Guerrero-Preston et al., 2014; Graves & Bauer, 2016; Kim & Chung, 2016). However, as a newly emerging approach, research of PAM is relatively limited. We aimed to identify transcriptional changes of SCC15 cells after PAM stimulation. We also hoped that the RNA-seq analysis could provide more information for intensive investigation.

In this study, next-generation mRNA sequencing (RNA-seq) technology was utilized to compose the transcriptomic changes in PAM-treated SCC15 cells, with the aim of understanding the biological mechanisms of alterations in cells induced by PAM. Oral squamous cell carcinoma cell line SCC15 was used in our study. Over 175 million short pair-end reads were generated and mapped to reference genomes. The subsequent functional analysis was performed to identify differentially expressed genes DEGs and regulatory pathways. QRT-PCR and Western blot were also conducted to validate the RNA-seq data. Gene Ontology (GO) analysis revealed that most of DEGs were categorized into “biological process”, suggesting that PAM induced an overall change of signal transduction in cancer cells. KEGG mapping and Gene Set Enrichment Analysis (GSEA) identified several pathways that were highly enriched, especially “p53 pathway”. However, SCC15 cells were commonly used as a p53-null cell line (Rheinwald & Beckett, 1981; Min et al., 1994). The p53 status of SCC15 cells used in our study was validated by qRT-PCR and Western blot. Therefore, the enriched p53 pathway-related genes in our analysis might be activated by other stimulators, in a p53-independent manner. It’s commonly recognized that “p53 pathway” may be activated in a p53-dependent manner. To avoid confusion, we use the term “p53 pathway” (with quotation marks) to refer to p53 pathway-related gene expression, representing an independently defined set of genes that are regulated by p53. In addition, some novel pathways, such as the hypoxia pathway, were also identified by GSEA. This study focused on genome-wide transcriptional alterations of PAM-treated cancer cells, providing not only genomic resources for future studies but also novel insights critical for the understanding of anti-cancer therapy of PAM.

### Materials and Methods

### Plasma device and PAM generation

The CAP device was a typical needle-ring plasma jet (Liu et al., 2016). The plasma jet is made of a polytetrafluoroethylene (PTFE) tube, a tungsten steel needle and a stainless steel ring. The PTFE tube has an inner diameter of 3 mm and the tungsten steel needle is coaxially placed in the center of the tube. The inner diameter of the stainless steel ring is 2 mm and is placed at the end of the PTFE tube. The distance between the tip of the needle and the ring electrode is 1 mm. The needle serves as high voltage (HV) electrode and the ring serves as the ground electrode. The HV electrode is connected to a DC power (purchased from Zhaofu Corp, Xian, China) via a 500 kΩ ballast resistor. When the air flows through the PTFE tube, a cold plasma plume is generated in the surrounding air. The plasma jet was used to vertically irradiate the media in a 6 cm plate. A total of 5 mL media were irradiated with the glow region of the plasma plume for 5 min (6 mm distance from the jet tip). Then, the media were balanced in cell incubator and used to stimulate SCC15 cells. The plume core (3 mm distance from the jet tip) temperature was detected under different conditions by Liu et al. (2016).

### Cells and PAM treatment

Human oral cancer cell line SCC15 cells (Cat.3111C0001CCC000524) were purchased from National Infrastructure of Cell Line Resource (http://www.cellresource.cn) and kept in our laboratory. SCC15 cells were generally cultured in Dulbecco’s modified Eagle’s medium (DMEM) (Life technologies, NY, USA) supplemented with 10% FBS, 50 µM β-ME, 100 U/ml penicillin, and 100 μg/ml streptomycin under the standard cell culture conditions (37 °C, 5% CO_2_ environment). 1.5 × 10^6^ SCC15 cells were seeded in 6 cm plates (Corning, NY, USA) the day before treatment. After 24 h culture, cells were washed with PBS 3 times. PAM was generated as mentioned above and added to the culture at a final volume of 5 mL. Then cells were incubated for 1 h at 37 °C. The medium was used as a control. The cells were washed and harvested with 1 mL Trizol reagent (Life technologies, NY, USA) by using a cell scraper. Then, the samples were quick-frozen in liquid nitrogen for RNA isolation.

### Cell viability assay

Cell Viability was measured with the Cell Counting Kit-8 (CCK-8; Transgen Biotech, Beijing, China) under the manufacturer’s instruction. In brief, 1.0 × 10^4^ SCC15 cells were seeded in 96-well plates the day before treatment. After 24 h culture, PAM was generated and added to each well at a final volume of 50 µL for the indicated times. Here we did a time course stimulation (0, 15, 30, 60, 120 min). To facilitate detection and promote consistency, we added the PAM in reverse order. After treatment, cells were washed and incubated with the CCK-8 reagent at the same time (90 µL fresh media plus 10 µL CCK-8). Then cells were incubated for 1 h and the absorbance of OD 450 nm was measured by using a Microplate Reader (SynergyHTX, BioTek, Winooski, VT, USA). The relative viability was calculated according to the manufacturer’s instructions.

### RNA isolation, cDNA library construction and RNA sequencing

Three independent experiments were performed for each group to obtain biological replicates. For sample preparation, 3 µg RNA per sample was used as input material. DNA contaminations were removed from the samples with DNase I. The purity, concentration and integrity of the RNA were assessed using a Nanodrop Spectrophotometer (Thermo Scientific, MA, USA) and an Agilent 2100 Bioanalyzer (Agilent Technologies, CA, USA). The sample libraries were constructed with Illumina TruSeq RNA Sample Preparation Kit (Illumina, CA, USA) according to the manufacturer’s instructions. The quantity and quality of the libraries were assessed using the Qubit Fluorometer (Life Technologies, CA, USA) and the Agilent Bioanalyzer 2100 System. The libraries were then sequenced using the Illumina HiSeq platform.

### Differential gene expression analysis

To obtain high-quality clean reads for subsequent analysis, adaptor reads, low-quality reads and reads with more than 10% unknown bases (poly-N) were removed from raw data. The Q30 and GC content of the clean data were also calculated. The clean reads were mapped to the human reference genome (GRCh37) using TopHat2 (Kim et al., 2013). The expression levels were calculated by using the fragments per kilobase per million reads method (FPKM) (Mortazavi et al., 2008). Differential gene expressions were analyzed by using the DEseq R package (Anders & Huber, 2010). The absolute value of Fold Change ≥2 and False Discovery Rate (FDR) ≤0.01 was adopted as the standard for determining the significance of differential gene expression.

### Functional analysis of DEGs

To identify functional categories of differentially expressed genes, Gene Oncology (GO) enrichment analysis was performed using the Database for Annotation, Visualization and Integrated Discovery (DAVID) (https://david.ncifcrf.gov/) (Huang da, Sherman & Lempicki, 2009b; Huang da, Sherman & Lempicki, 2009a). Following the instructions of the DAVID manual, DEGs were uploaded and the function charts were generated. The groups with a *P*-value <0.05 and gene counts more than two were examined. KEGG pathway enrichment analysis of DEGs was performed using KOBAS software (Xie et al., 2011). The rich factor is calculated as the ratio of the numbers of DEGs enriched in this pathway, to the numbers of all genes annotated in the same pathway. The *Q*-value is the corrected *P*-value with threshold <0.05. Gene Set Enrichment Analysis (GSEA) was performed using the Java GSEA implementation in our study (http://software.broadinstitute.org/gsea/index.jsp) (Subramanian et al., 2005). The gene lists of KEGG or hallmark gene signature were adopted from The Molecular Signatures Database (MSigDB). As a metric for ranking genes in GSEA, the absolute signal to noise value of gene expression was used, and other parameters were set to default values.

### Quantitative RT-PCR

Cells were processed and harvested under the same conditions as the sequencing sample. Total RNA was isolated by using Trizol reagent and was reverse-transcribed with the PrimeScript RT Kit (TAKARA Biotech, Dalian, China). The qRT-PCR assays were conducted on the Bio-Rad CFX96 System using the TransStart Tip reagent (TransGen Biotech). Primers used in the present study were designed by using Primer premier software (Table S1). Relative mRNA levels were calculated by the comparative cycle threshold method and GAPDH was used as the internal control for each sample.

## Results

### CAP device and PAM treatment

**Figure 1:**
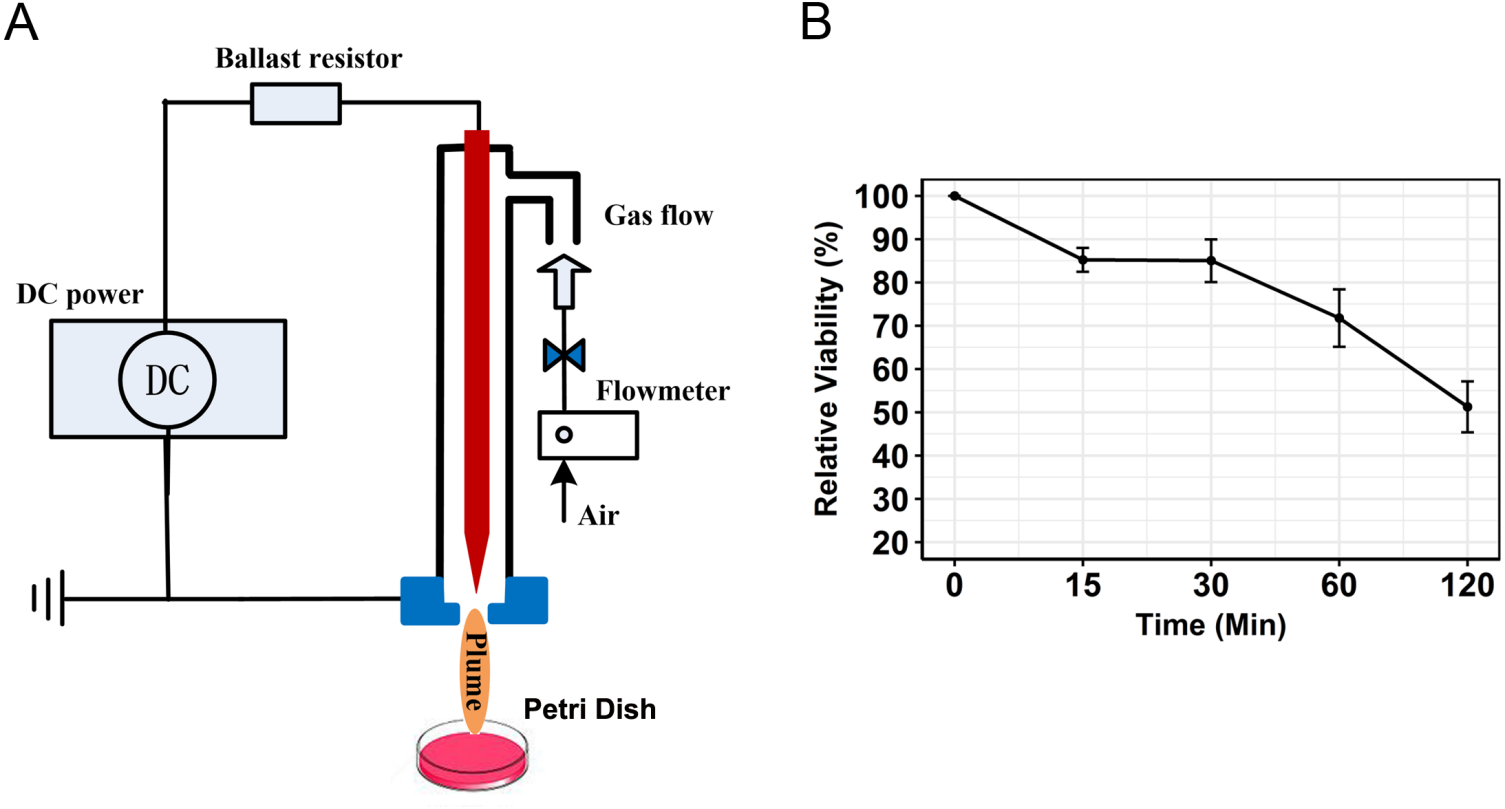
Schematic diagram of the CAP and viability of SCC15 cells treated with PAM. (A) The generation of PAM by a typical CAP device. DMEM were placed in a plate and activated by CAP. (B) PAM showed strong anti-cancer capacity on SCC15 cells. SCC15 cells were treated with PAM at indicated times. Cell viability decreased with increasing incubation time of PAM.

The CAP device is a typical needle-ring plasma jet designed by Liu et al. (2016). Ambient air was used as the working gas, which has been used by several groups in anti-cancer research (Kim et al., 2010a; Kim et al., 2010b; Arndt et al., 2013). A schematic diagram of the system is presented in Fig. 1A. Cold plasma was generated when air flowed through the PTFE tube. The air was supplied by a pump and the flow rate controlled by gas flowmeter at a rate of 3 L/min, plus the input voltage of DC power was −5 KV. PAM was generated by using a general strategy. The plasma jet was used to vertically irradiate the media in a 6 cm plate. After irradiation, the media were used to stimulate cancer cells.

To characterize the kinetics of PAM-treated cancer cells, relative viabilities were detected by the CCK-8 method. For 15 min treatment, PAM was able to stimulate a decrease of cell viability of target SCC15 cells (85.22 ± 2.8 %) (Fig. 1B). Relative viability was kinetically augmented with increasing incubation time of PAM (30 min, 85.0 ± 4.9%; 60 min, 71.8 ± 6.6%; 120 min, 51.3 ± 5.9%). The results suggested that PAM showed a noticeable anti-cancer ability on SCC15 cancer cells. These data were representatives of at least three separate experiments. To elucidate the early transcriptional changes, sequencing samples were prepared in 6 cm plates for 60 min incubation with PAM.

### RNA sequencing and read mapping

We established 6 libraries with the following designations. PAM1, PAM2 and PAM3 were from PAM-treated SCC15 cells while Ctr1, Ctr2 and Ctr3 were mock-treated with three biological replicates respectively. The cDNA libraries were prepared and sequenced by Illumina HiSeq platform, which generated a total of 175,673,694 raw reads (Table S2A). After removing the adaptors and low-quality reads, we obtained 172,801,869 clean reads, with a high quality of Q30 ≥ 88.98%. We then mapped the trimmed clean reads onto the human genome and 75.21% to 79.59% of the clean reads were mapped uniquely to the genome, whereas a small percentage of them were mapped multiple times onto the genome (Table S2B). The whole subsequent analysis was based on the uniquely mapped reads.

### Differentially expressed genes (DEGs) in response to PAM

**Figure 2:**
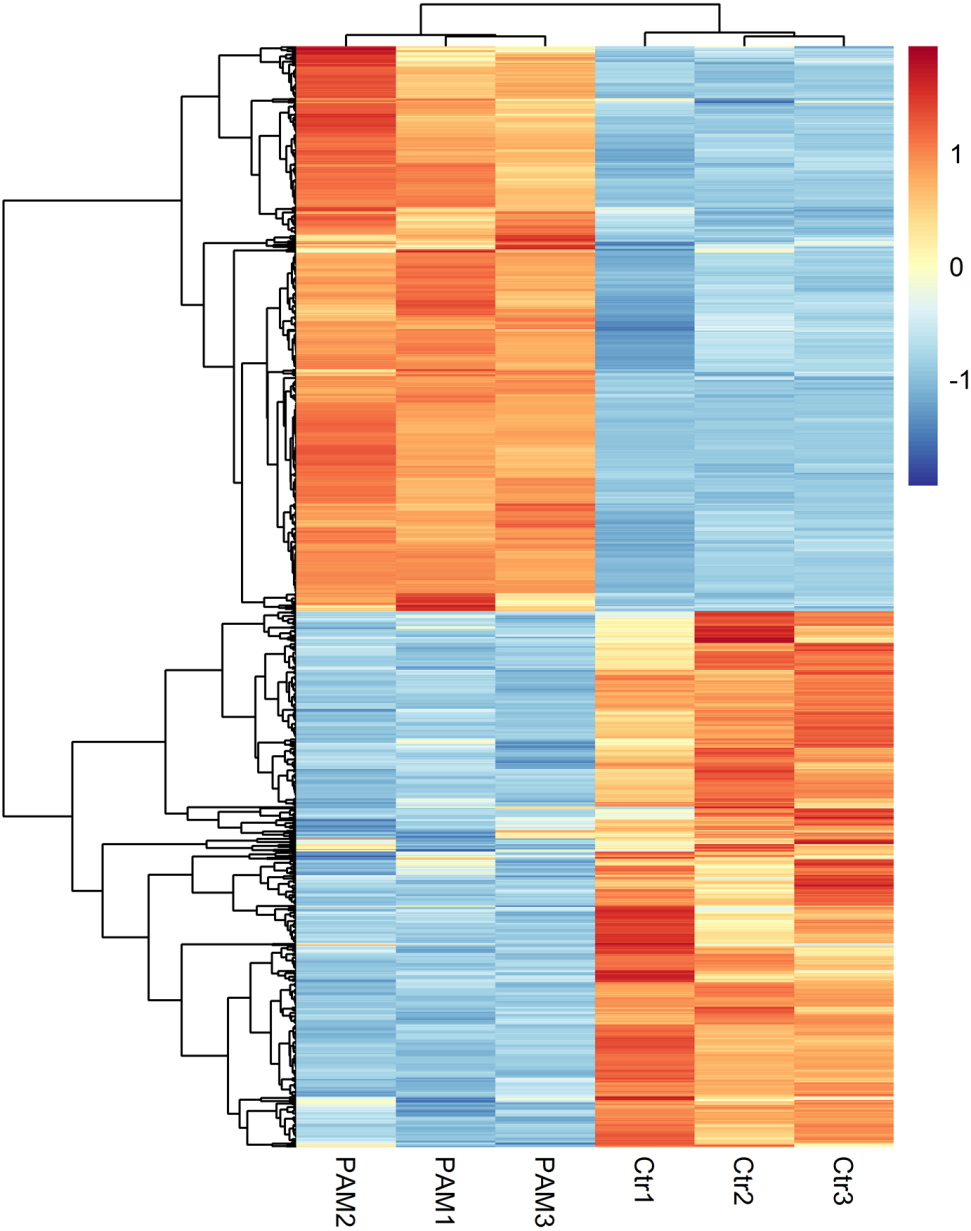
Global changes of DEGs across all samples. Hierarchical clustering of DEGs was generated after PAM treatment. The expression levels were visualized and the scale from least abundant to highest range is from −2.0 to 2.0. Their phylogenetic relationships were shown on the left tree. The top tree indicated the cluster relationship of the samples.

The levels of gene expression were expressed as FPKM values and displayed a comparable distribution among 6 parallel libraries. A total of 15,446 genes were annotated in this study and are available in Table S3. The biological replicates of both control and PAM-treated samples exhibited high correlation (Pearson’s Correlation Coefficient R2 >0.81) in FPKM values (Fig. S1). DEseq was employed to screen for DEGs and a total of 934 annotated genes were identified to be differentially expressed when considering exclusively a stringent threshold of FDR ≤ 0.01 and fold-change ≥ 2 (Table S4). To obtain a global view of these DEGs, hierarchical clustering was constructed with normalized FPKM values to characterize changes across six samples (Fig. 2). The most up-regulated gene was Glutathione-specific gamma glutamylcyclotransferase 1 (CHAC1, 16-fold), a pro-apoptotic component of the unfolded protein response, suggesting the involvement of apoptotic pathway after PAM stimulation.

### Gene ontology (GO) classifications

**Figure 3:**
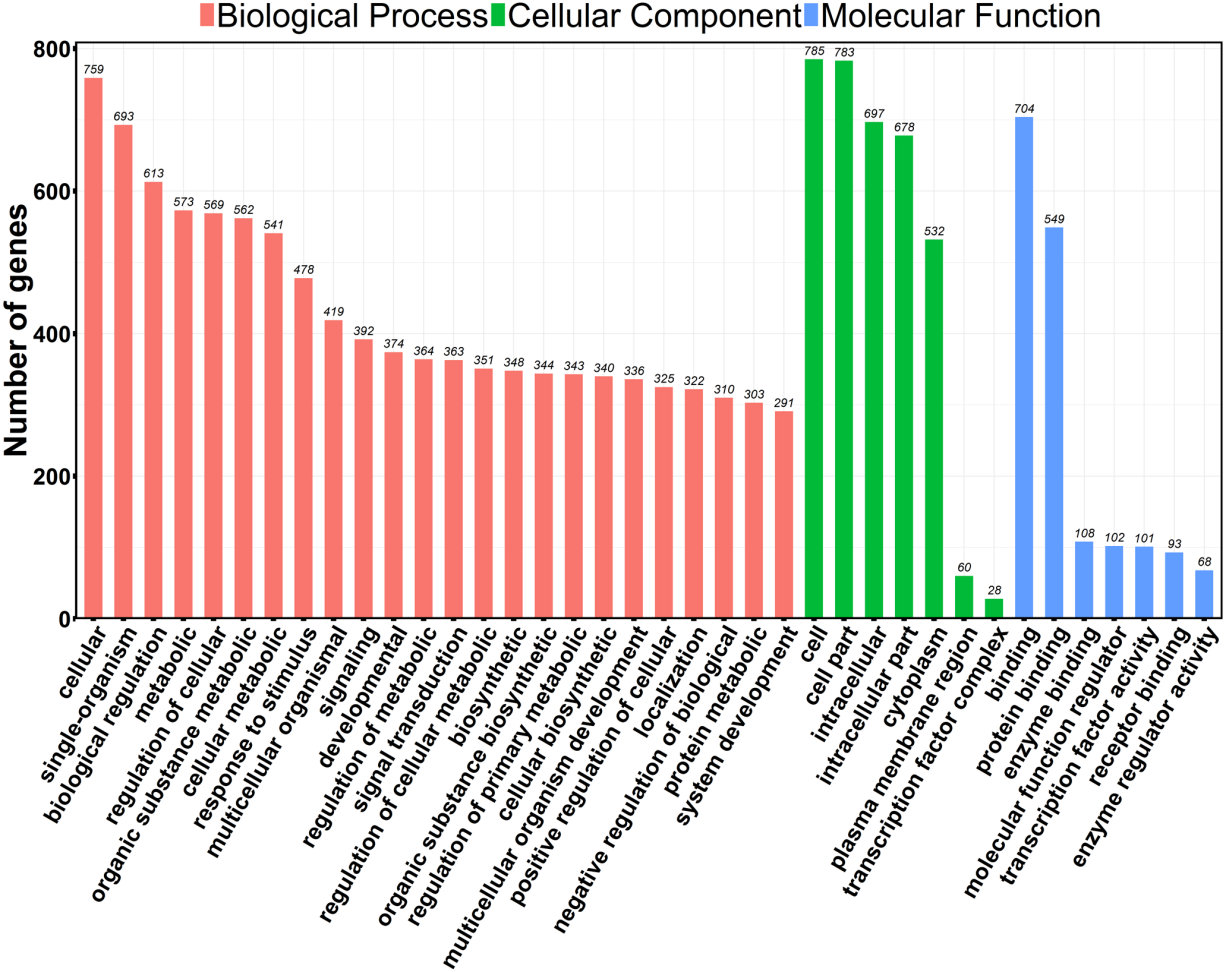
GO functional classification of the DEGs. The distributions are summarized in three main categories: biological process, molecular function (MF), and cellular component (CC). The *x*-axis indicates different GO terms and the *y*-axis indicates the number of genes in each category.

GO is a universally acknowledged gene functional enrichment database and is generally applied to search for enriched GO terms in DEGs (Ashburner et al., 2000). We performed GO analysis using web-based DAVID tool (Huang da, Sherman & Lempicki, 2009b; Huang da, Sherman & Lempicki, 2009a). 845 out of 934 profiled DEGs were assigned to 1,211 GO terms, including 1,101 biological processes, 16 cellular components and 94 molecular function terms. Typical enriched GO terms are shown in Fig. 3. The GO terms of molecular function category were concentrated in “binding” (704 DEGs, 75.4%) and “protein binding” (549 DEGs, 58.8%) The highest percentages of GO terms under cellular component class were “cell” (785 DEGs, 84.0%) and “cell part” (783 DEGs, 83.8%). We noted that GO terms were mainly categorized into “biological processes”, with wide distributions and extensive assignments. The most prevalent “biological processes” assignment was “cellular”, including 759 DEGs and accounting for 81.3% of all DEGs. Besides, some important assignments, such as “biological regulation” (582 DEGs, 62.3%), “metabolic” (573 DEGs, 61.3%) and “signaling” (392 DEGs, 42.0%), were highly enriched, suggesting that the biological processes of cancer cells were widely changed after PAM treatment. The data of GO classifications are available in Table S5.

### “P53 pathway” was highly enriched by KEGG mapping

**Figure 4:**
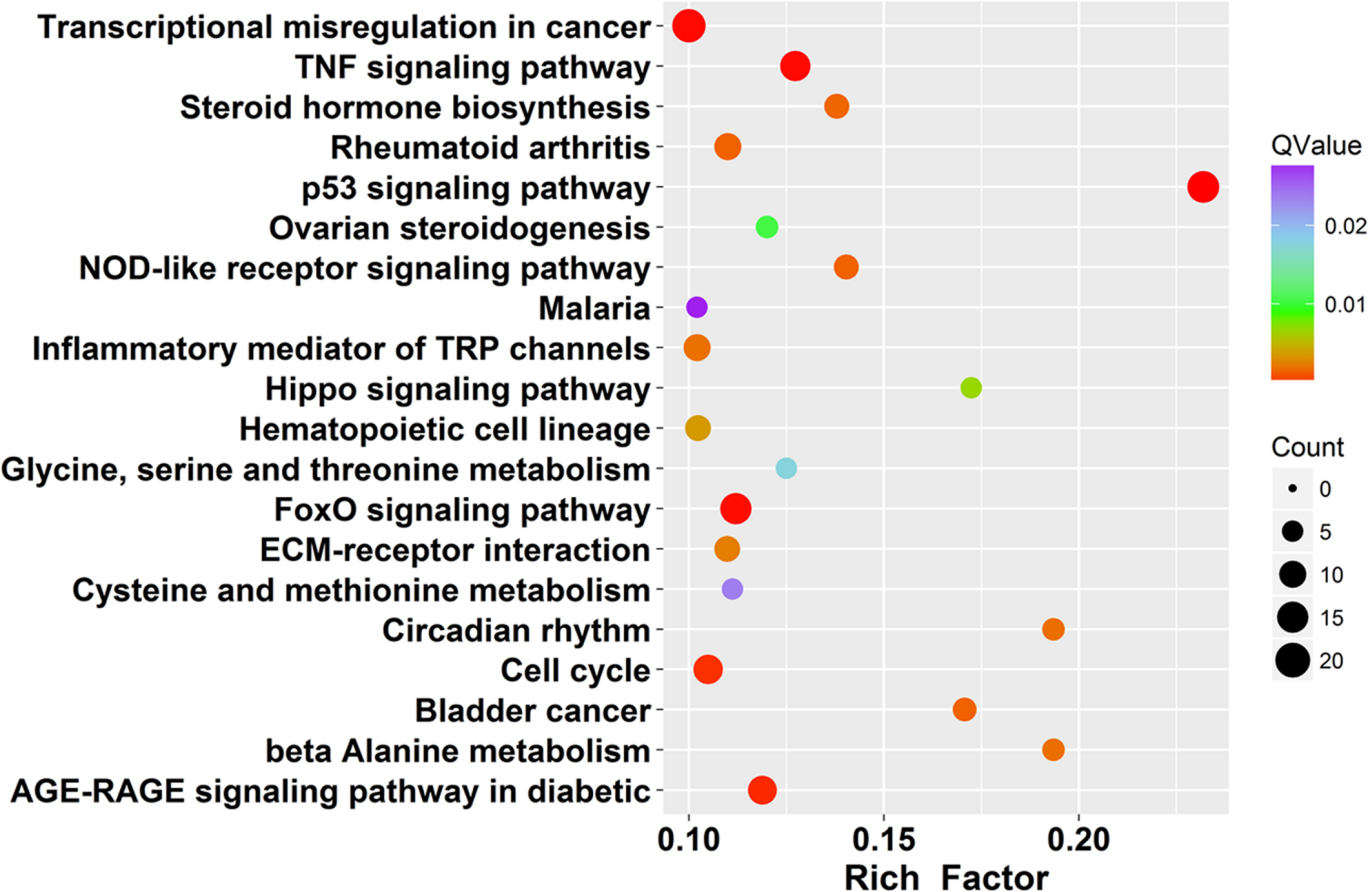
Scatter plot of enriched KEGG pathways statistics. Rich factor is the ratio of the differentially expressed gene number to the total gene number in a certain pathway. *Q*-value is corrected *P*-value ranging from 0 ∼ 1. The color and size of the dots represent the range of the *Q*-value and the number of DEGs mapped to the indicated pathways, respectively. Top 20 enriched pathways are shown in the figure.

KEGG database is a collection of various pathways, representing the molecular interactions and reaction networks (Kanehisa et al., 2004). To identify signaling pathways involved in PMA-treated cells, we had mapped the KEGG database and found that identified DEGs were significantly enriched in 40 KEGG pathways (*Q* value ≤ 0.01, Table S6). The top 20 enriched pathways are shown in Fig. 4. DEGs were highly clustered in several signaling pathways, such as “p53 signaling”, “Hippo signaling”, “TNF signaling”, “AGE-RAGE signaling” and “FoxO signaling”, suggesting that PAM may perform its function through these pathways.

**Figure 5:**
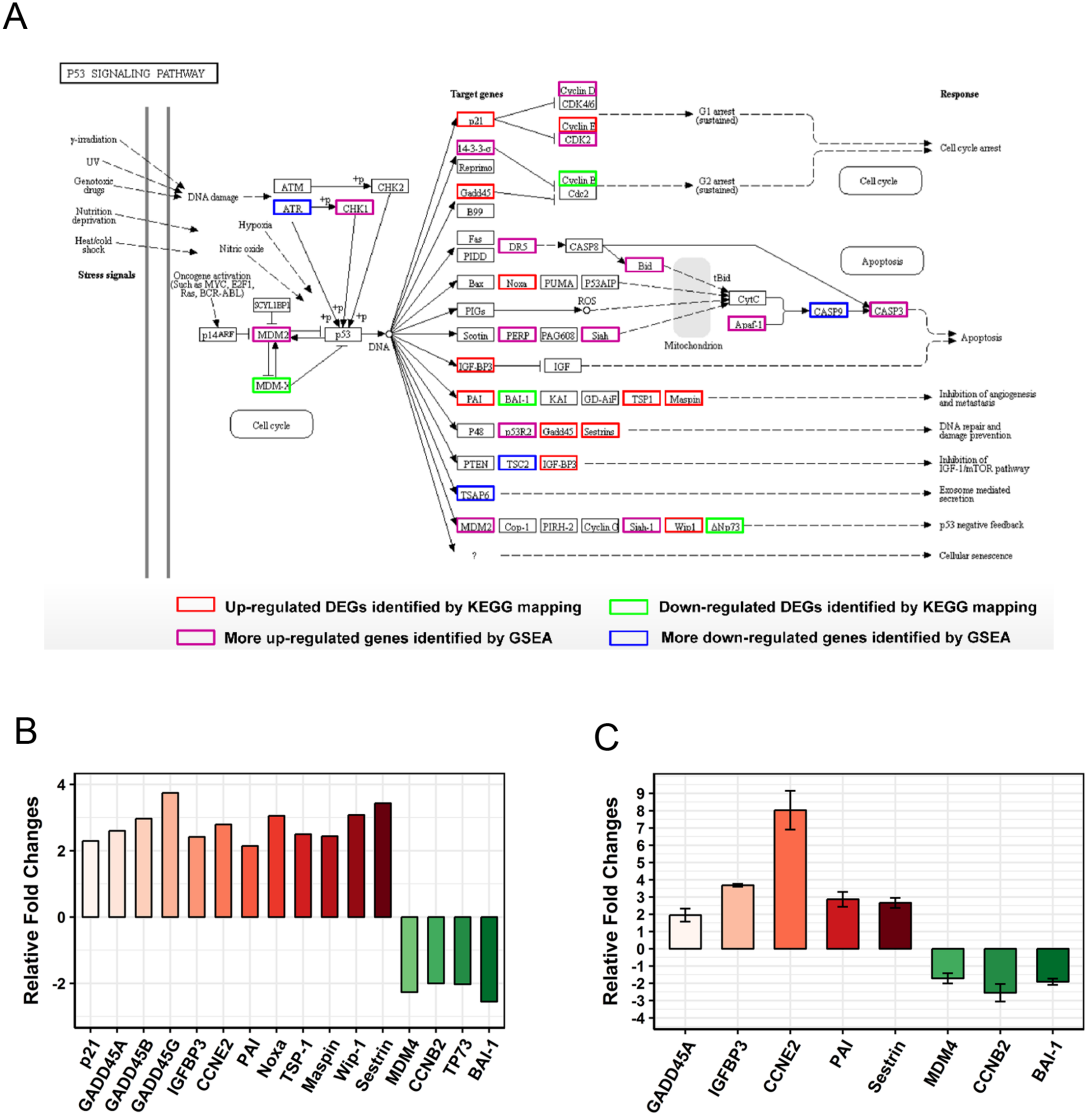
PAM remodeled the gene expression profile of the “p53 pathway”. (A) The map of “p53 pathway” was modified from the KEGG map. The red boxes indicated up-regulated DEGs and green boxes indicated down-regulated DEGs identified by KEGG mapping. The purple boxes (up-regulated) and blue boxes (down-regulated) were identified by GSEA. (B) The relative fold changes of 16 DEGs of RNA-seq data. (C) Expression levels of selected genes in “p53 pathway” are examined by qRT-PCR. Results are presented as the mean ± s.d. of three repeated experiments.

**Figure 6:**
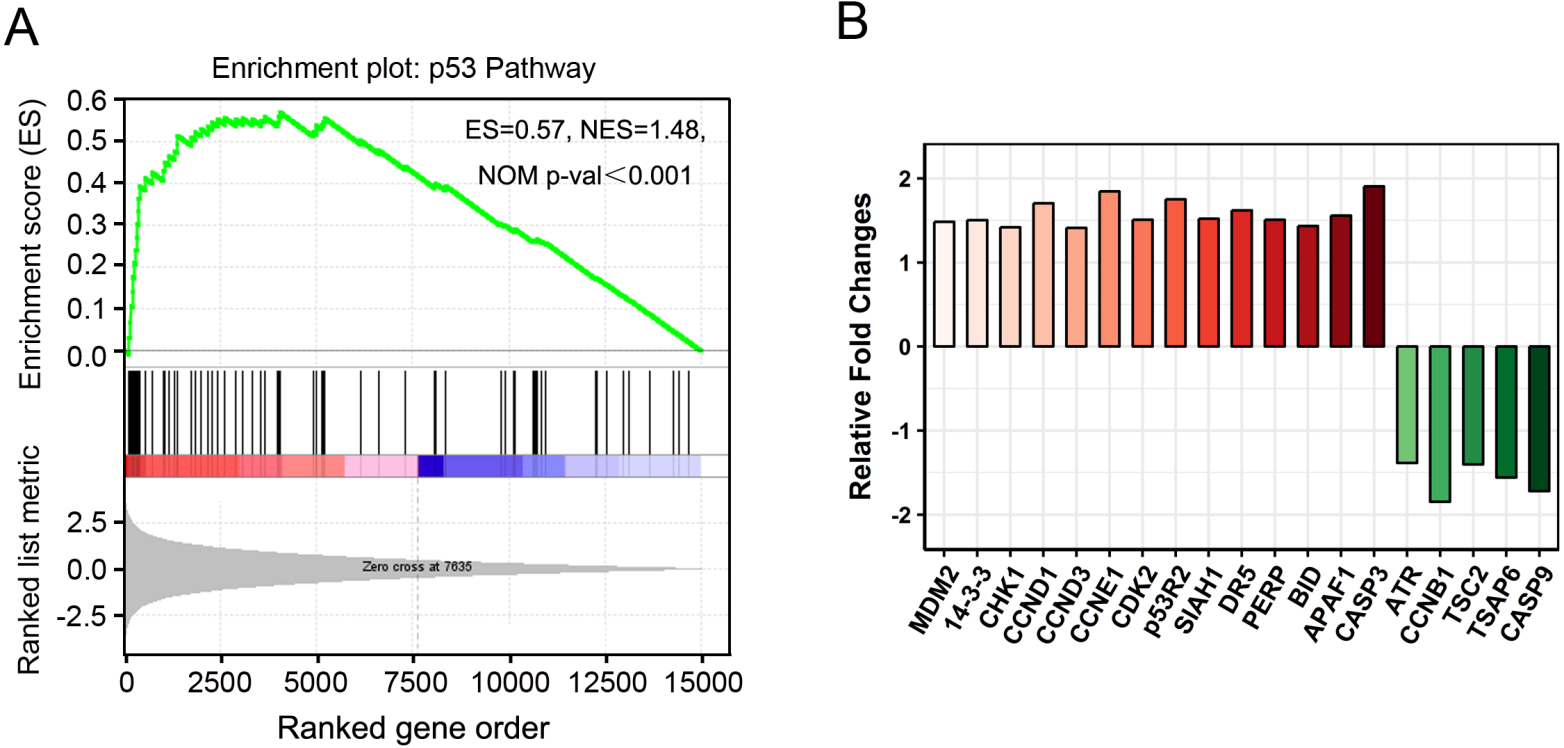
“P53 pathway” clustered by gene set enrichment analysis (GSEA). (A) GSEA results showing “p53 pathway” signatures enriched in PAM-treated cancer cells. ES, enrichment score; NES, normalized ES; NOM *p*-val, normalized *p*-value. (B) The relative fold changes of 19 genes in “p53 pathway” identified by GSEA.

The “p53 pathway” was identified as the most significantly enriched pathway in KEGG analysis (Rich Factor = 0.23 and *Q* value < 0.001). A total of 16 DEGs were enriched in this pathway (Fig. 5A). Moreover, 12 genes (*GADD45A*, *SERPINE1*, *SERPINB5*, *PPM1D*, *IGFBP3*, *SESN2*, *CCNE2*, *GADD45B*, *THBS1*, *GADD45G*, *CDKN1A*, *PMAIP1*) were up-regulated and 4 genes (*MDM-4*, *CCNB2*, *TP73*, *ADGRB1*) were down-regulated (Fig. 5B). To further confirm these observations, eight genes were selected for qRT-PCR validation (Fig. 5C). The results of the qRT-PCR analysis were also consistent with our RNA-seq data and validate its reliability. In addition, we detected CCNB2 and Apaf1 expression by Western blot (Fig. S2). CCNB2 is a member of the cyclin family and is essential for cell cycle regulation. Apaf1 is the key component of apoptosome and acts in both p53 and apoptosis pathways. The mRNA expression levels of CCNB2 and Apaf1 were regulated in SCC15 cells after PAM stimulation (Figs. 5 and 6). We incubated SCC15 cells with PAM for different times and validated that the protein levels of CCNB2 and Apaf1 were changed, which were in agreement with our previous results.

### Gene set enrichment analysis (GSEA)

GSEA is a useful computational algorithm that determines whether a pre-defined set of genes (signature) shows differences between two groups (Mootha et al., 2003; Subramanian et al., 2005). To gain further insights at the gene-set level, GSEA was performed against KEGG or hallmark gene-set signature. Consistently, “p53 pathway” was found to be enriched in PAM phenotype (Fig. 6A). Importantly, instead of 16 DEGs identified by KEGG analysis, 19 more genes with fewer changes (Fold Change ≤ 2) were found in GSEA (Figs. 6B, 5A). In addition, apoptosis and hypoxia pathways were also identified as the principal involved pathways in this assay, although they were not enriched in KEGG analysis (Fig. S3 and Table S7). Our results not only establish the valuable application of GSEA in expression analysis but also provide novel insights into cancer cell signaling induced by PAM.

## Discussion

In the present study, transcriptome sequencing and differential gene expression analysis were performed on PAM-treated human oral SCC15 cells. PAM is an alternative application of CAP and has attracted wide attention in recent years (Tanaka et al., 2011; Yan et al., 2015; Boehm et al., 2016; Chen et al., 2016). Compared to traditional CAP, PAM can be stored for a long time and used without the CAP device (Adachi et al., 2015; Judée et al., 2016). DMEM plus 10% FBS was used as the source solution of PAM in our study, without the addition of antibiotics or β-ME (Yan et al., 2014; Boehm et al., 2016; Yan et al., 2016a; Yan et al., 2016b). In fact, some additives such as ferrum and lysine were proved to enhance the anti-tumor ability of PAM (Yan et al., 2016a; Adachi et al., 2016).

Recently, there were several reports about the CAP/PAM induced transcriptional changes of normal tissue or cancer cells. Yokoyama, Johkura & Sato (2014) compared the plasma-medium with H_2_O_2_-medium in stimulating HeLa cells with microarray. They found that the expression pattern was different between the two treatments. Rosani et al. (2015) directly irradiated human cornea tissues with CAP and performed RNA-seq analysis. In addition, Schmidt et al. (2016) explored the different expression genes of Jurkat cells and THP-1 cells using microarray approaches . They found that the gene expression levels of anti-oxidative defense and redox regulation were significantly changed. Our present study was mainly focused on the overall changes and signaling pathways of PAM-stimulated oral SCC15 cells, trying to provide more information for the understanding of the anti-tumor effect of PAM.

This study demonstrated that EggNOG functional clustering showed that 243 DEGs can be classified into “signal transduction mechanisms” (Fig. S4). GO-based analysis was consistent with this observation and further revealed that most of DEGs were categorized into “biological process” (Fig. 3 and Table S5). Gene ontology (GO) classifies each gene according to its attributes (function or location) (Ashburner et al., 2000). GO analysis is useful for us to get an overall view of PAM-induced differential gene expression. For example, there were 127 DEGs identified under the GO category “GO: 0042981∼regulation of apoptotic process”, while only 11 genes were identified by KEGG analysis and 65 genes were identified by GSEA in apoptosis pathway (Tables S6 and S7). More genes with apoptotic function identified by GO analysis would provide us more information for further study. Figure 3 shows the 1st levels of GO classification and Table S5 lists all GO results.

KEGG mapping implicated that several signaling pathways were highly enriched, including “p53 pathway”, “Hippo pathway”, “TNF pathway”, “AGE-RAGE pathway” and “FoxO pathway” (Fig. 4 and Table S6). In addition, GSEA was performed and suggested that not only “p53 pathway” but also hypoxia and apoptosis pathways were enriched (Fig. S3). Compared with the extensive studies of direct CAP treatment, the signaling pathways of PAM stimulation remains limited. The mode of cell death induced by PAM has been investigated by several groups (Adachi et al., 2015; Hirokazu et al., 2015; Saito et al., 2016). Their research showed that PAM could effectively induce tumor cell death, in spite of the plasma devices or gas types. The central player in the death pathway was mitochondria, which could be destroyed by reactive species of PAM. Several apoptotic proteins were identified to be activated during this process, such as AIF, PARP-1 and Drp1 (Adachi et al., 2015; Hirokazu et al., 2015; Saito et al., 2016). In our study, 65 genes in apoptosis pathway were identified by GSEA, providing more candidates for further study.

The “p53 pathway” was identified as the most significantly enriched pathway in KEGG analysis. This observation was confirmed by GSEA analysis and 19 more DEGs were identified (Fig. 6). It’s interesting that oral SCC15 cells were reported to have low levels of *TP53* mRNA and have undetectable levels of p53 protein (Rheinwald & Beckett, 1981; Min et al., 1994). To validate the p53 status, we performed qRT-PCR and Western blot to detect p53 in SCC-15 cell line used in our study. We found that the mRNA level of *TP53* was low and p53 protein was undetectable even for a long time exposure (Fig. S5). In addition, PAM stimulation had no effect on the p53 status of both mRNA and protein levels. However, our RNA-seq data showed activation of p53 pathway-related genes. We also performed qRT-PCR and Western blot to validate the expression of some of these genes (Fig. 5 and Fig. S2). A question was raised as to how these genes are regulated in p53 null cells? The most likely reason was the presence of p53 family members such as p63 and p73 (Pflaum, Schlosser & Müller, 2014). The p63 and p73 proteins had several isoforms with different functions, including tumour-suppressor or oncogenic effects (Orzol et al., 2015). Some of the isoforms could act similar to p53 protein and play a redundant role (Pflaum, Schlosser & Müller, 2014). In addition, there is plenty of other evidence to suggest that p53 pathway-related genes could be regulated in a p53-independent manner, mediated by C/EBP, Rb, Oct1/NF-Y, or MELK respectively (Chinery et al., 1997; Sun et al., 1998; Hirose et al., 2003; Matsuda et al., 2017). It was shown previously that cellular ROS up-regulated the expression of p21 in a p53-independent manner (Russo et al., 1995). The observation implicated that plasma could generate ROS and might act in this way. Considering that CAP and PAM both act depending on reactive species, the study of CAP will also facilitate the understanding of PAM. Kim et al. (2011) showed that CAP activated p53 and induced p53-dependent apoptosis. Ma et al. (2014) reported that cancer cells carrying a mutated *TP53* gene were more vulnerable to CAP treatment than cells with wild-type p53, implicating p53-independent cell death caused by CAP. These observations suggested that plasma-induced cell death could be p53-dependent or p53-independent, which is consistent with our knowledge (Liebermann, Hoffman & Steinman, 1995).

## Conclusions

In summary, this study has presented RNA-seq based transcriptomic analysis of PAM-treated SCC15 cells. Our results have demonstrated that several important signaling pathways, such as “p53 pathway”, might play critical roles during PAM treatment. Although the precise mechanisms that are involved remain largely unknown, this study has expanded our understanding systematically and provided a foundation to further study the application of PAM in anti-cancer therapy.

##  Supplemental Information

10.7717/peerj.3751/supp-1 Figure S1 Pearson’s Correlation Coefficient of RNA-seq results

10.7717/peerj.3751/supp-2 Figure S2 PAM stimulation induced changes of CCNB2 and Apaf1 on protein levels 1.5 × 10^6^ SCC15 cells were seeded in 6 cm plates and cultured 24 h. Cells were mock-treated or treated by PAM for indicated times (1, 2, 4 hour each) and harvested for immunoblot analysis. The expression of CCNB2 was down-regulated (A) and Apaf1 was up-regulated (B). GAPDH was used as a loading control.

10.7717/peerj.3751/supp-3 Figure S3 Novel pathways clustered by GSEA Both hypoxia (A) and apoptosis (B) related genes were significantly clustered by GSEA, while not enriched by KEGG mapping.

10.7717/peerj.3751/supp-4 Figure S4 EggNOG functional clustering of DEGs

10.7717/peerj.3751/supp-5 Figure S5 The mRNA and protein expression of p53 gene of SCC15 cell line 1.5 × 10^6^ SCC15 cells were seeded in 6 cm plates and cultured 24 hours. Cells were mock-treated or treated by PAM for 1 h. Total RNA and proteins were extracted and applied for QRT-PCR or Western blot analysis. (A) The mRNA level of p53 was low. Results are presented as the mean ±s.d. of three repeated experiments. (B) The p53 protein was undetectable in SCC15 cells. The middle panel showed no p53 expression even at a long time exposure of the membrane. HEK293T cells were used as a positive control to confirm the experimental system and GAPDH was used as a loading control.

10.7717/peerj.3751/supp-6 Table S1 The primers used in qRT-PCR analysis

10.7717/peerj.3751/supp-7 Table S2 Summary of RNA-seq Summary of sequence assembly after Illumina sequencing (A) and clean reads mapped to the reference genome (B).

10.7717/peerj.3751/supp-8 Table S3 The total detected genes in the RNA-seq data The raw RNA-seq data was processed and the expression was calculated by using the fragments per kilobase per million reads method (FPKM). PAM1, PAM2 and PAM3 were from PAM-treated SCC15 cells while Ctr1, Ctr2 and Ctr3 were mock-treated with 3 biological replicates respectively.

10.7717/peerj.3751/supp-9 Table S4 The differentially expressed genes (DEGs) identified in our analysis DEGs were analyzed by using the DEseq R package. The absolute value of Fold Change ≥2 and False Discovery Rate (FDR) ≤ 0.01 were adopted as the criteria for determining the significance of differential gene expression.

10.7717/peerj.3751/supp-10 Table S5 GO results of DEGs (A) The Gene Ontology (GO) biological process enrichment. (B) The Gene Ontology (GO) cellular component enrichment. (C) The Gene Ontology (GO) molecular function enrichment.

10.7717/peerj.3751/supp-11 Table S6 Summary of pathways enriched in KEGG mapping

10.7717/peerj.3751/supp-12 Supplemental Information 1 Genes identified by GSEA analysis Genes of “p53 pathway”, apoptosis pathway and hypoxia pathway were identified by GSEA analysis.

10.7717/peerj.3751/supp-13 Supplemental Information 2 Supplementary Materials and Methods

## Additional Information and Declarations

### Data Availability

The following information was supplied regarding data availability: The Genome Sequence Archive (GSA): PRJCA000445.
