# Supplementary figures and images for: Gene expression profiling and functional analysis reveals that p53 pathway-related gene expression is highly activated in cancer cells treated by cold atmospheric plasma-activated medium
Source: PeerJ. 2017 Aug 25;5:e3751. doi: 10.7717/peerj.3751 (PMC5572956; doi:10.7717/peerj.3751)

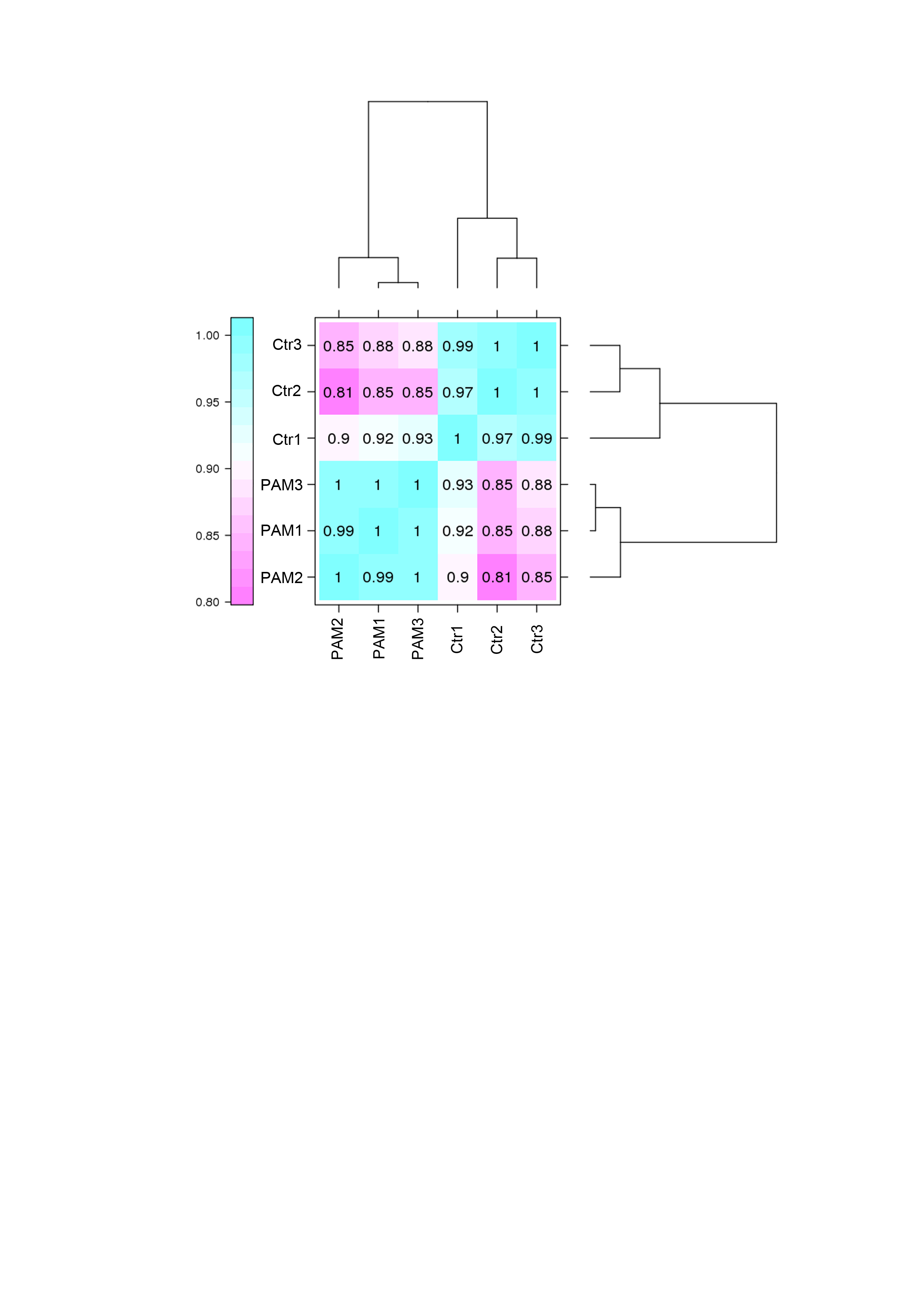

Supplement: Figure S1 [file peerj-05-3751-s001.png]

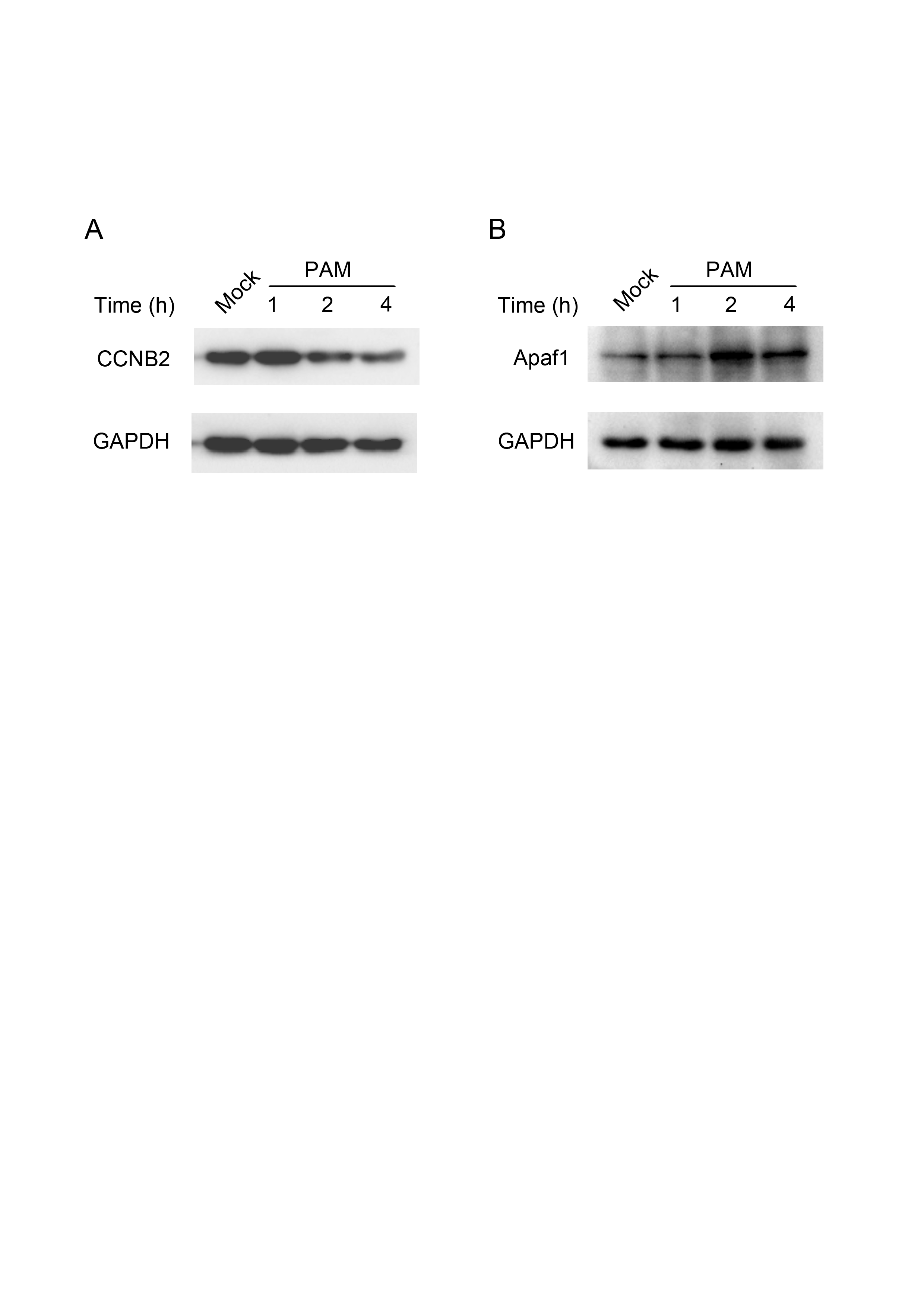

Supplement: Figure S2 — 1.5 × 10^6^ SCC15 cells were seeded in 6 cm plates and cultured 24 h. Cells were mock-treated or treated by PAM for indicated times (1, 2, 4 hour each) and harvested for immunoblot analysis. The expression of CCNB2 was down-regulated (A) and Apaf1 was up-regulated (B). GAPDH was used as a loading control. [file peerj-05-3751-s002.png]

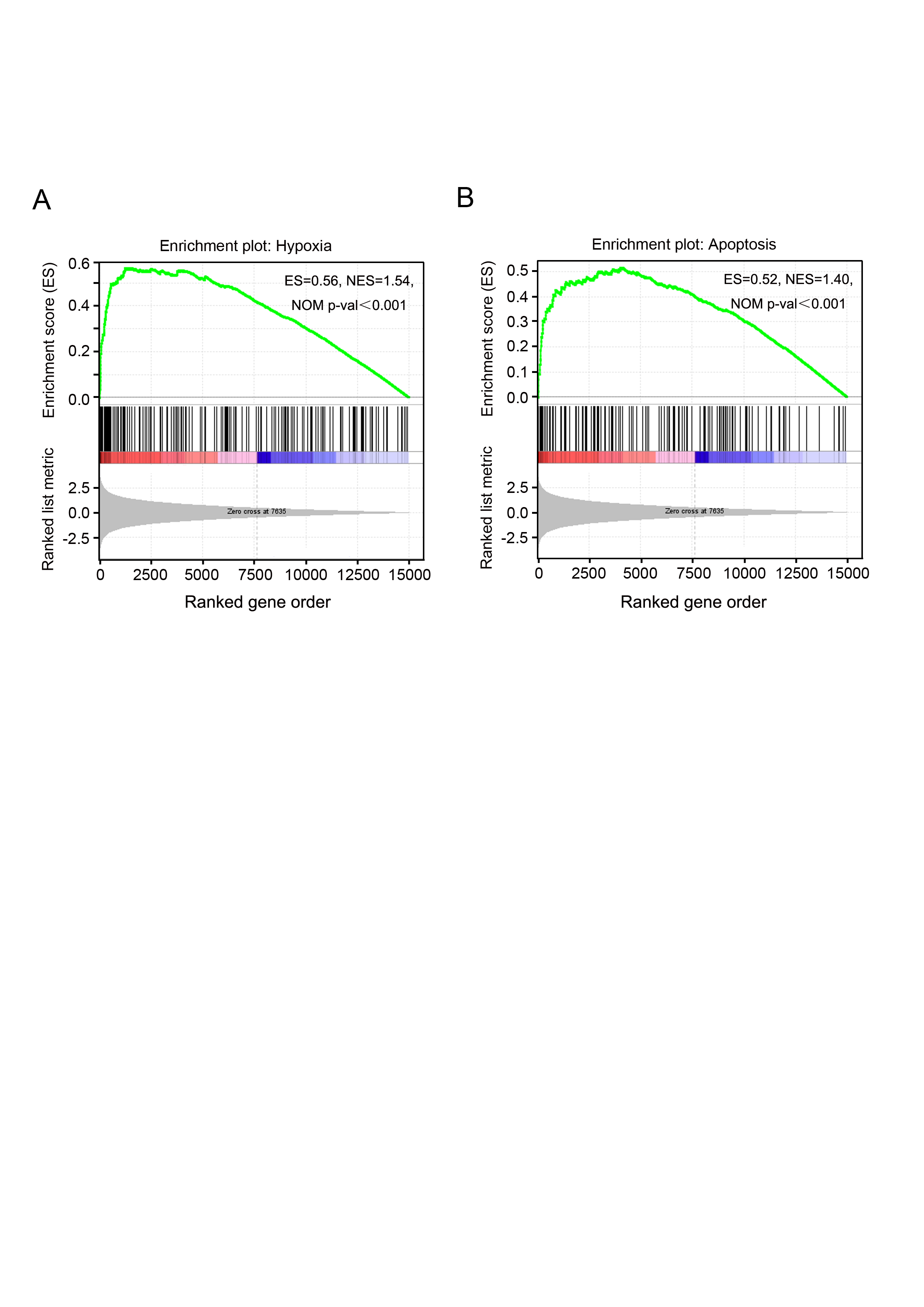

Supplement: Figure S3 — Both hypoxia (A) and apoptosis (B) related genes were significantly clustered by GSEA, while not enriched by KEGG mapping. [file peerj-05-3751-s003.png]

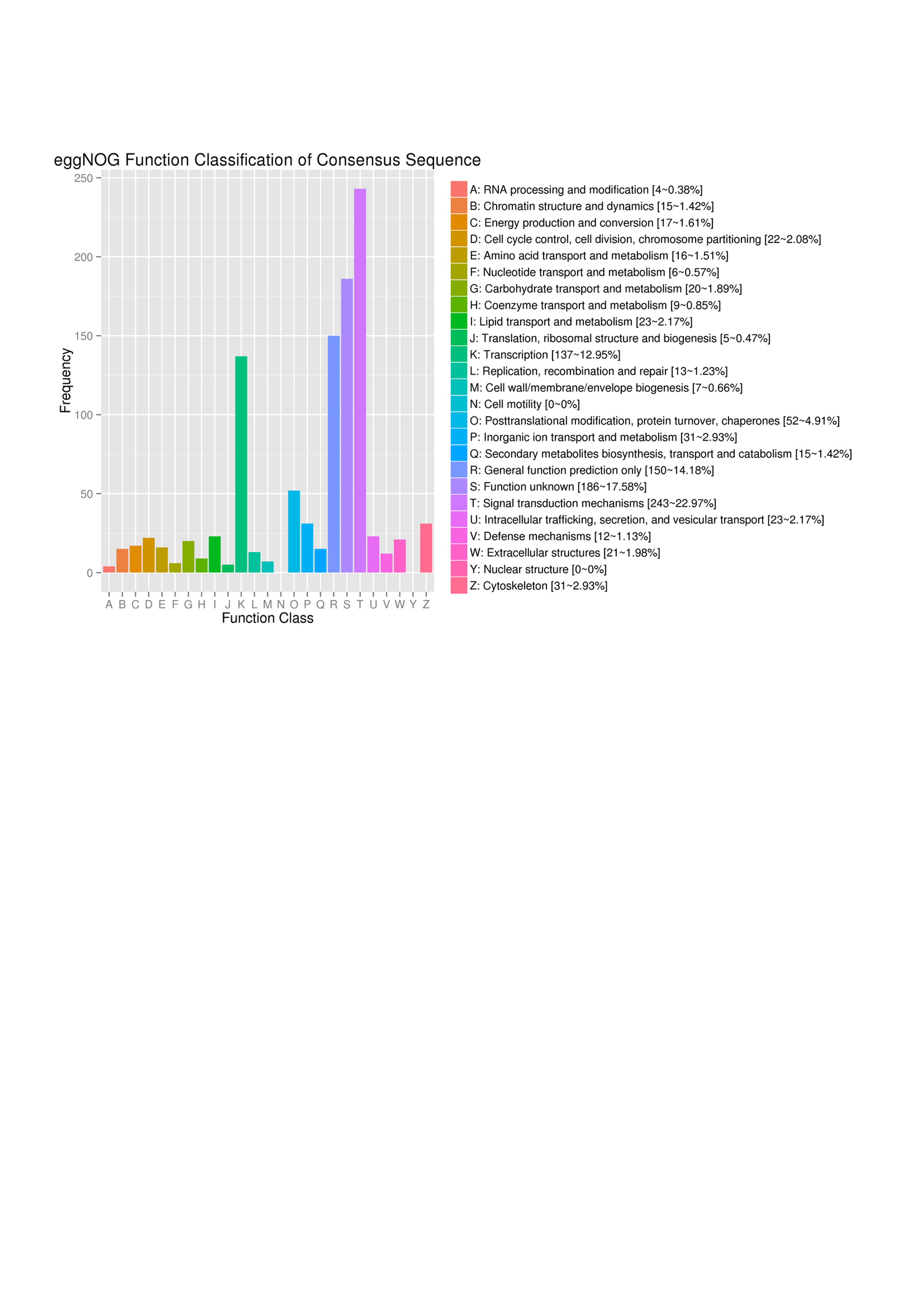

Supplement: Figure S4 [file peerj-05-3751-s004.png]

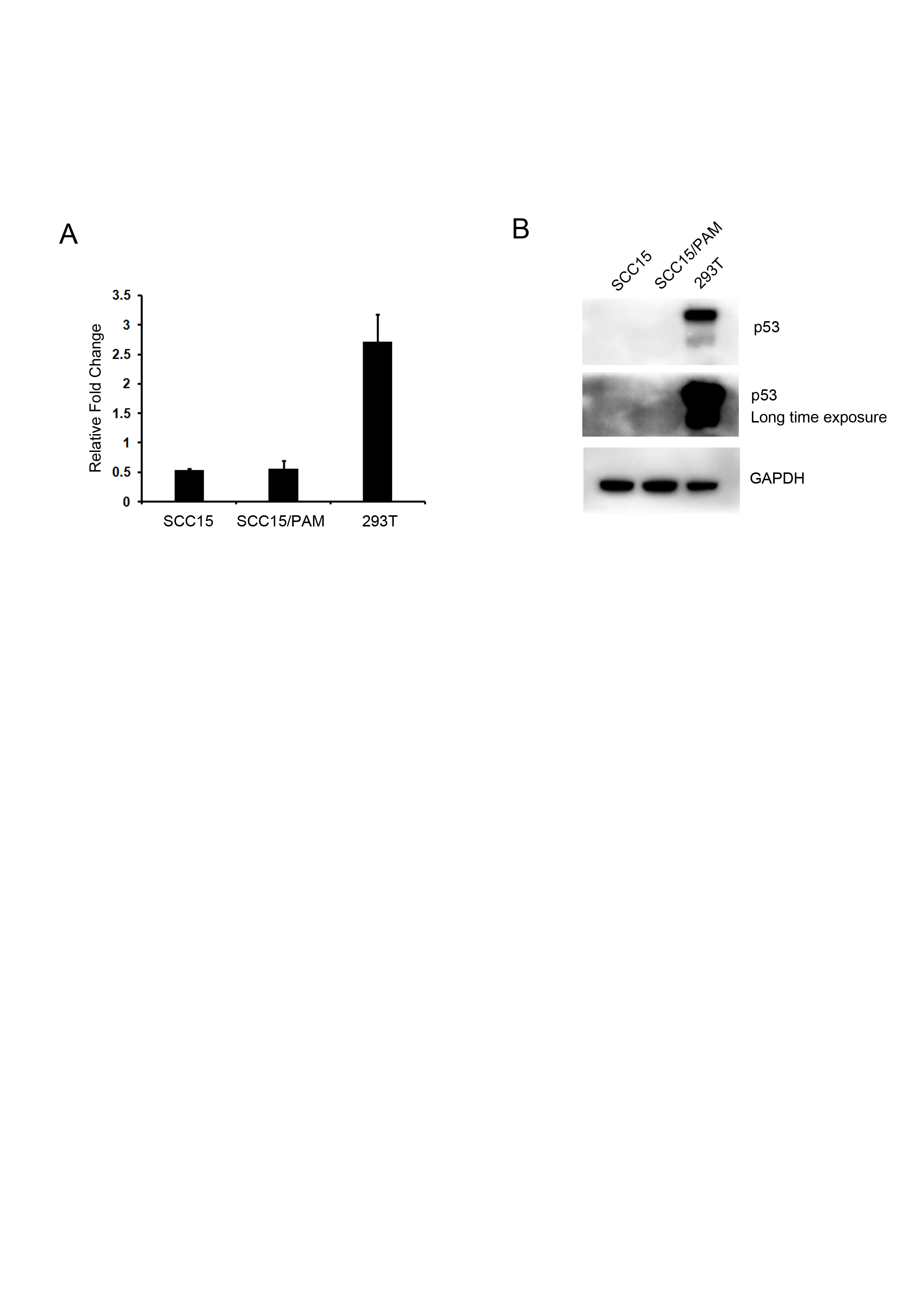

Supplement: Figure S5 — 1.5 × 10^6^ SCC15 cells were seeded in 6 cm plates and cultured 24 hours. Cells were mock-treated or treated by PAM for 1 h. Total RNA and proteins were extracted and applied for QRT-PCR or Western blot analysis. (A) The mRNA level of p53 was low. Results are presented as the mean ±s.d. of three repeated experiments. (B) The p53 protein was undetectable in SCC15 cells. The middle panel showed no p53 expression even at a long time exposure of the membrane. HEK293T cells were used as a positive control to confirm the experimental system and GAPDH was used as a loading control. [file peerj-05-3751-s005.png]
