# Supplementary material for: Gene expression profiling and functional analysis reveals that p53 pathway-related gene expression is highly activated in cancer cells treated by cold atmospheric plasma-activated medium
Source: PeerJ. 2017 Aug 25;5:e3751. doi: 10.7717/peerj.3751 (PMC5572956; doi:10.7717/peerj.3751)
Supplement: Table S1 [file peerj-05-3751-s006.doc]

**Supplementary Table 7 Primers used for real-time PCR.**

| **Gene** | **Entrez ID** | **Forward (5’-3’)** | **Reverse (5’-3’)** |
| --- | --- | --- | --- |
| GADD45A | 1647 | GAGAGCAGAAGACCGAAAGGA | CACAACACCACGTTATCGGG |
| IGFBP3 | 3486 | CCCTGCCGTAGAGAAATGGAA | GCCCATACTTATCCACACACCA |
| CCNE2 | 9134 | CAGGTTTGGAGTGGGACAGTAT | ACTTCCTCCAGCATAGCCAAA |
| PAI | 5054 | CTCTCTGCCCTCACCAACATT | TCGGTCATTCCCAGGTTCTCT |
| Sestrin | 83667 | CCGCCATCAGTGTTCTTACCT | GTGTTCCTTGGTGATGAGCCA |
| MDM4 | 4194 | TTTTGATCCCTGCAACTCAGTG | GTCTCGTGGTCTTTTCTCACAT |
| CCNB2 | 9133 | CCTAGTGGATTGGCTGGTACA | GCCAAGAGCAGAGCAGTAATC |
| BAI-1 | 575 | GTGGAGGTACATTCGCTCAGA | AGCAGAAGGAGGACAGGAAGA |
| GAPDH | 2597 | GGAGCGAGATCCCTCCAAAAT | GGCTGTTGTCATACTTCTCATGG |
