# Supplementary material for: Gene expression profiling and functional analysis reveals that p53 pathway-related gene expression is highly activated in cancer cells treated by cold atmospheric plasma-activated medium
Source: PeerJ. 2017 Aug 25;5:e3751. doi: 10.7717/peerj.3751 (PMC5572956; doi:10.7717/peerj.3751)
Supplement: Table S2 — Summary of sequence assembly after Illumina sequencing (A) and clean reads mapped to the reference genome (B). [file peerj-05-3751-s007.doc]

| Supplementary Table 1A Summary of sequence assembly after Illumina sequencing | | | | | |
| --- | --- | --- | --- | --- | --- |
| Sample ID | Raw reads | Clean reads | Clean bases | %≥Q30 | GC Content |
| Ctr1 | 27410436 | 27166052 | 8038851466 | 88.98% | 53.99% |
| Ctr2 | 33829790 | 31814579 | 9436166730 | 90.14% | 54.89% |
| Ctr3 | 33147480 | 32939782 | 9754503754 | 89.80% | 54.55% |
| PAM1 | 28610228 | 28462162 | 8377266438 | 89.60% | 54.49% |
| PAM2 | 27130871 | 26999886 | 8019925640 | 88.98% | 54.84% |
| PAM3 | 25544889 | 25419408 | 7531576844 | 89.32% | 54.44% |
| *Ctr1- Ctr3: Control; PAM1- PAM3: Plasma-treated cell samples;* | | | | | |
| *Q30: The percentage of bases with a Phred value > 30.* | | | | | |

Supplementary Table 1B Summary of clean reads mapped to the reference genome

| Sample ID | Total Reads | Mapped Reads | Unique Map Reads | Multiple Map Reads |
| --- | --- | --- | --- | --- |
| Ctr1 | 54332104 | 44799335 (82.45%) | 42520034 (78.26%) | 2279301 (4.20%) |
| Ctr2 | 63629158 | 53525603 (84.12%) | 47853776 (75.21%) | 5671827 (8.91%) |
| Ctr3 | 65879564 | 55327249 (83.98%) | 52435750 (79.59%) | 2891499 (4.39%) |
| PAM1 | 56924324 | 47304135 (83.10%) | 45291659 (79.56%) | 2012476 (3.54%) |
| PAM2 | 53999772 | 44876001 (83.10%) | 42598298 (78.89%) | 2277703 (4.22%) |
| PAM3 | 50838816 | 42951745 (84.49%) | 39925059 (78.53%) | 3026686 (5.95%) |

*Ctr1- Ctr3: Control; PAM1- PAM3: Plasma-treated cell samples.*
