# Supplementary material for: Gene expression profiling and functional analysis reveals that p53 pathway-related gene expression is highly activated in cancer cells treated by cold atmospheric plasma-activated medium
Source: PeerJ. 2017 Aug 25;5:e3751. doi: 10.7717/peerj.3751 (PMC5572956; doi:10.7717/peerj.3751)
Supplement: Supplemental Information 2 [file peerj-05-3751-s013.doc]

**Supplementary Materials and Methods**

**Cells, antibodies and reagents**

Human Embryonic Kidney (HEK) 293T cells were obtained from the Cell Bank of Type Culture Collection of Chinese Academy of Sciences (Shanghai, China) and cultured in DMEM medium supplemented with 10% FBS, 50 μM β-ME, 100 U/ml penicillin, and 100 μg/ml streptomycin under the standard cell culture conditions (37°C, 5% CO_2_ environment). Commercial antibodies were rabbit pAb against GAPDH (D110016, BBI, Sangon), mouse anti-p53 (DO-7, Santa Cruz), rabbit anti-Apaf1 (380882, Zen Bio), rabbit anti-CCNB2 (381384, Zen Bio), HRP-conjugate secondary antibodies (ZSGB Bio). Primers specific for p53 gene were listed as following. P53 sense, 5'- CAGCACATGACGGAGGTTGT -3' and antisense, 5'- TCATCCAAATACTCCACACGC -3'.

**Western blot analysis**

1.5 x 10^6^ SCC15 or HEK293T cells were seeded on 6 cm plate the day before treatment. After 24 hours culture, cells were or mock-treated or treated by PAM for indicated times. Total proteins were extracted by 100 μL lysis buffer (20 mM Tris-HCl, pH 8.0, 150 mM NaCl, 1 mM EDTA, 0.5% NP-40, 1 mM DTT and protease inhibitor cocktail). Samples (20 μL of each) were loaded and separated by SDS-PAGE, then transferred onto PVDF membranes and immunoblotted with the indicated antibodies.
